# A QBO Cookbook: Sensitivity of the Quasi‐Biennial Oscillation to Resolution, Resolved Waves, and Parameterized Gravity Waves

**DOI:** 10.1029/2021MS002568

**Published:** 2022-03-22

**Authors:** Chaim I. Garfinkel, Edwin P. Gerber, Ofer Shamir, Jian Rao, Martin Jucker, Ian White, Nathan Paldor

**Affiliations:** ^1^ Fredy and Nadine Herrmann Institute of Earth Sciences Hebrew University Jerusalem Israel; ^2^ Courant Institute of Mathematical Sciences New York University New York USA; ^3^ Key Laboratory of Meteorological Disaster Ministry of Education (KLME) Joint International Research Laboratory of Climate and Environment Change (ILCEC) Collaborative Innovation Center on Forecast and Evaluation of Meteorological Disasters (CIC‐FEMD) Nanjing University of Information Science and Technology Nanjing China; ^4^ Climate Change Research Centre and ARC Centre of Excellence for Climate Extremes University of New South Wales Sydney Australia

**Keywords:** quasi‐biennial oscillation, tropical stratosphere, gravity waves

## Abstract

An intermediate complexity moist general circulation model is used to investigate the sensitivity of the quasi‐biennial oscillation (QBO) to resolution, diffusion, tropical tropospheric waves, and parameterized gravity waves. Finer horizontal resolution is shown to lead to a shorter period, while finer vertical resolution is shown to lead to a longer period and to a larger amplitude in the lowermost stratosphere. More scale‐selective diffusion leads to a faster and stronger QBO, while enhancing the sources of tropospheric stationary wave activity leads to a weaker QBO. In terms of parameterized gravity waves, broadening the spectral width of the source function leads to a longer period and a stronger amplitude although the amplitude effect saturates in the mid‐stratosphere when the half‐width exceeds ∼25m/s. A stronger gravity wave source stress leads to a faster and stronger QBO, and a higher gravity wave launch level leads to a stronger QBO. All of these sensitivities are shown to result from their impact on the resultant wave‐driven momentum torque in the tropical stratosphere. Atmospheric models have struggled to accurately represent the QBO, particularly at moderate resolutions ideal for long climate integrations. In particular, capturing the amplitude and penetration of QBO anomalies into the lower stratosphere (which has been shown to be critical for the tropospheric impacts) has proven a challenge. The results provide a recipe to generate and/or improve the simulation of the QBO in an atmospheric model.

## Key Points

- Sensitivity of the quasi‐biennial oscillation (QBO) to resolution, dissipation, wave forcing, and parameterized gravity waves is explored in a single framework
- The influence of these factors on the QBO can be related to their impact on wave‐induced momentum fluxes in the deep tropics
- The QBO period can be tuned independently of its amplitude, but the vertical structure (particularly at lower levels) is harder to capture

## 1 Introduction

The dominant mode of variability in the tropical stratosphere, the quasi‐biennial oscillation, consists of downward propagating easterly and westerly wind regimes, with a period typically ranging from 24 to 32 months (Baldwin et al., 2001). Although the QBO is a tropical phenomenon, it impacts the atmospheric circulation and composition globally through a variety of mechanisms. One of the earliest remote influences to be recognized is the so‐called “Holton‐Tan effect” whereby the QBO modulates the strength of the stratospheric polar vortex (Anstey & Shepherd, 2014; Garfinkel et al., 2012; Holton & Tan, 1980; Rao et al., 2020b), and this effect is projected to intensify under climate change (Rao et al., 2020c). The QBO also directly influences tropospheric variability by affecting the Pacific subtropical jet (Garfinkel & Hartmann, 2011a, 2011b) and tropical convection on both seasonal mean (Collimore et al., 2003; Liess & Geller, 2012; Rao et al., 2020a) and subseasonal timescales (Martin et al., 2019; Yoo & Son, 2016; Zhang & Zhang, 2018). QBO signals are also evident in temperature and in stratospheric constituents such as ozone and water vapor (Diallo et al., 2018; Randel & Wu, 1996; Randel et al., 1998; Tian et al., 2019).

The QBO is driven by waves propagating upwards from the troposphere with periods unrelated to (and much faster than) that of the resulting oscillation. Lindzen and Holton (1968) showed how a QBO could be driven by a broad spectrum of vertically propagating waves (with phase speeds in both westward and eastward directions), in which a two‐way feedback between the waves and the background flow leads to oscillating winds. The first part of the feedback is that the background flow modulates the propagation and damping/dissipation of the waves. The second part of the feedback is that when the waves experience damping or dissipation, they flux momentum to the background flow. Holton and Lindzen (1972) and Plumb (1977) demonstrated that only two wave modes (one with easterly and one with westerly phase speeds) are required as long as dissipation of waves occurs near, and not solely at, the critical lines. An important implication of this earlier work is that the period and amplitude of the oscillation are controlled, in part, by the spectral range and amplitude of the momentum fluxed by these waves. The particular waves associated with the QBO was the focus of later work, and both large‐scale waves (especially Kelvin waves for the westerly regime) and smaller scale gravity waves have been found to be crucial (Ern et al., 2014; Pahlavan et al., 2021).

While a few models began to successfully simulate a spontaneous QBO‐like oscillation some 20 years ago (Hamilton et al., 2001; Scaife et al., 2000; Takahashi, 1996, 1999), only around five models participating in Coupled Model Intercomparison Project Phase 5 (CMIP5) spontaneously simulated it, and the majority of CMIP6 models still have no QBO (Rao et al., 2020a, 2020b; Richter et al., 2020). Even in CMIP models that succeed in simulating a QBO with period and amplitude relatively close to that observed, the QBO winds suffer from an inability to propagate downwards to the lower stratosphere, a bias also evident in models participating in the quasi‐biennial oscillation initiative (QBOi; Bushell et al., 2020). Furthermore, the representation of the waves that fundamentally drive the QBO differs dramatically among the QBOi models (Holt et al., 2020), with, for example, Kelvin wave activity barely evident in some models while too strong in others. Diversity in the representation of mixed Rossby‐gravity waves, which also contributes to the driving of the QBO, is even more pronounced (Holt et al., 2020). The models with stronger convectively coupled waves rely less heavily on zonal mean forcing from parameterized gravity waves (Holt et al., 2020). All but one of these models (the MIROC model) also includes a parameterization of gravity waves (Bushell et al., 2020), as the resolved waves are apparently not energetic enough to force the QBO at resolutions typically used by these models.

The QBO is sensitive not only to the generation of resolved wave modes, but also to their subsequent upwards propagation. Some of the resolved waves have a characteristic vertical wavelength of a few kilometers (Figures 8 and 10 of Kiladis et al., 2009), and hence a model with, say, a vertical resolution of a kilometer (which is typical of CMIP and QBOi models in the lowermost stratosphere, Butchart et al., 2018) will not be able to accurately represent its upward propagation. The net effect is that the resolved wave forcing that reaches the QBO region, and hence the QBO itself, is influenced by vertical resolution (Anstey et al., 2016; Geller et al., 2016). Indeed, Holt et al. (2016) explored a model with 7 km horizontal resolution that included a realistic resolved wave spectrum and plentiful small‐scale gravity waves in the troposphere, but still required parameterized gravity waves due to a poor representation of resolved wave dissipation in the shear zones, due in part to the relatively coarse vertical resolution. The fact that at least 20 different CMIP and QBOi models still simulate a reasonable QBO reflects the fact that these models tune the parameterized gravity waves so that the overall momentum forcing is sufficient.

The goal of this study is to identify and isolate the role of resolution, dissipation, resolved wave forcing, and parameterized wave forcing, for the QBO. While many of these sensitivities have been reported before, here we assess a broader range of sensitivities all within a single modeling framework, with the expectation that results in our framework may be relevant to other models. Our hope is that these results can be used to more intelligently tune other models.

While it is possible to consider these factors in a multi‐model ensemble such as QBOi or CMIP6, the wide diversity in the representation of these factors among the models limits the confidence with which one can ascribe changes to a given cause. For example, the tropical climatology in comprehensive GCMs is (with good reason) made as realistic as possible, which necessarily limits the ability to examine how changing resolved waves impacts the QBO. It is also very difficult to perturb the resolution of a comprehensive model without severely altering its climatology, given the need to retune other scale‐sensitive parameterizations.

After describing the model and the gravity wave scheme in Section 2, we document the sensitivity to resolution, the gravity wave scheme, the hyperdiffusion, and the resolved waves in Section 3. We then explain how these various perturbations to the model lead to changes in QBO periodicity and downward propagation to the lower stratosphere in Section 4. We summarize our results and conclude with an example use of the cookbook to improve the QBO of our control integration in Section 5.

## 2 A Model of an Idealized Moist Atmosphere (MiMA)

We use the model of an idealized moist atmosphere (MiMA) introduced by Jucker and Gerber (2017), Garfinkel et al. (2020a), and Garfinkel et al. (2020b). This model builds on the aquaplanet models of Frierson et al. (2006), Frierson et al. (2007), and Merlis et al. (2013). Very briefly, the model solves the moist primitive equations on the sphere, employing a simplified Betts‐Miller convection scheme (A. Betts & Miller, 1986; A. K. Betts, 1986), idealized boundary layer scheme based on Monin‐Obukhov similarity theory, a slab ocean, and the Rapid Radiative Transfer Model (RRTMG) radiation scheme (Iacono et al., 2000; Mlawer et al., 1997). Please see Jucker and Gerber (2017) and Garfinkel et al. (2020b) for more details. Orography, ocean zonal heat transport, and land‐sea contrast (i.e., difference in heat capacity, surface friction, and moisture availability between oceans and continents) are specified as in Garfinkel et al. (2020b).

The details of the gravity wave scheme (developed by Alexander & Dunkerton [1999]) are included in Appendix A. Unless otherwise indicated, all simulations in this study were run with a triangular truncation at wavenumber 42 (T42; equivalent to a roughly 2.8° grid) with 40 vertical levels and a model top at 0.18 hPa, for 38 years after discarding at least 10 years as spinup. Vertical levels in the lower stratosphere and tropical tropopause layer are located at sigma levels 0.135, 0.112, 0.092, 0.076, 0.062, and 0.051, which leads to a resolution of ∼1.2 km.

**Figure 1:**
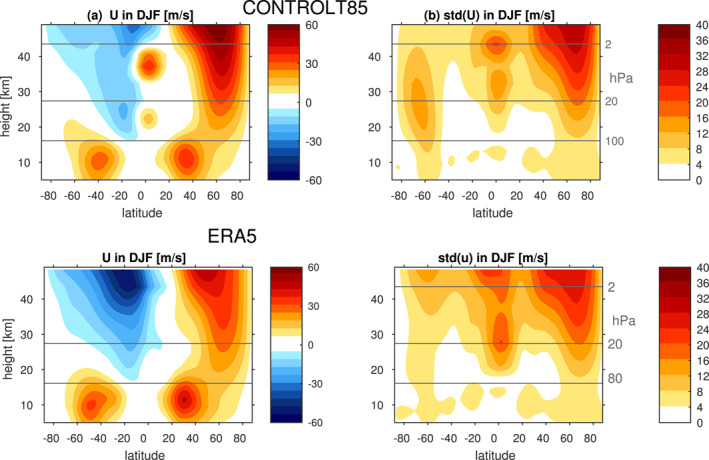
(a) Zonal mean zonal wind climatology in December through February; (b) standard deviation of the zonally averaged zonal wind. For (a), the contour interval is 6 m/s and the 0 m/s contour is omitted. (Top) in control at T85 with 40 vertical levels; (bottom) in ERA5.

This specification allows for a reasonable mean state in the model. Figure 1a shows the December to February climatology of the zonal winds in a control simulation (hereafter CONTROL) at T85 resolution, and Figure 1b shows the standard deviation of the winds. The model simulates a reasonable stratospheric and tropospheric mean state, and robust variability in the tropical stratosphere. The mean state in the tropical stratosphere suffers from a westerly bias which is even more severe at coarser resolution, however, and this leads to the QBO in our model suffering from a too strong westerly regime, and concomitantly, too weak an easterly regime. Gupta et al. (2020) found that such a bias occurs more commonly in spectral cores, as compared to, say, finite volume. Such a bias is also evident in some of the QBOi models examined by Bushell et al. (2020, see their Figure 2) and CMIP6 models examined by Rao et al. (2020b, see their Figure 1). Future work should confirm whether the sensitivities found here are robust in a model which does not suffer from this bias. Finally, midlatitude stationary waves, tropical precipitation, and stratospheric variability in CONTROL were found to be captured as well as many CMIP models (Garfinkel et al., 2020a, 2020b; Garfinkel, White, et al., 2021; White et al., 2020). As shown later, the model represents tropical wave modes realistically as well.

**Figure 2:**
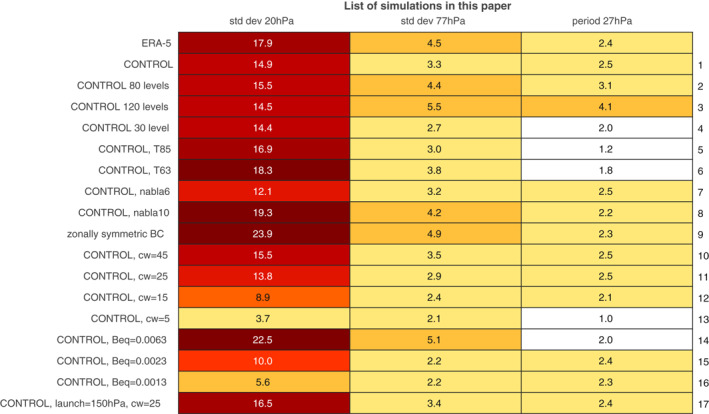
A list of experiments included in this study. Note that in addition to these 16 simulations, the scatter plots show additional integrations used in Garfinkel et al. (2020a) and Garfinkel et al. (2020b). Experiment 1 was performed at T42 with 40 vertical levels, $\nabla^{8}$ hyperdiffusion, cw = 35 m/s, $B_{eq}$ = 0.0043 Pa, and a launch height of 315 hPa, and the other experiments use these settings except as otherwise specified. For ERA‐5, the standard deviation at 80 hPa is shown instead of 77 hPa, and the period is computed at 30 hPa instead of 27 hPa. Note that while the T42L40 simulations simulate too weak a standard deviation at 20 hPa, they simulate too strong a standard deviation at 10 hPa. Cells are shaded to visually accent larger values.

We focus on the sensitivity of these key metrics of the QBO: the vertical structure of its amplitude, quantified by the standard deviation of zonal mean zonal winds at 20 hPa and at 77 hPa, representing the mid‐ and lower stratosphere respectively, and the periodicity, quantified by the peak power of the Fourier transformed zonal mean zonal wind at 27 hPa. These three definitions can be used even in cases with a poorly defined QBO, unlike definitions which explicitly quantify wind maxima. All of these metrics are computed after first applying a low‐pass ninth‐order Butterworth filter with a cutoff at 120 days in order to remove high frequency wave‐driven variability. The simulations performed, and the value of these metrics for each simulation, are listed in Figure 2. Note that the correlation between the amplitude at 27 hPa and the period across all simulations is small (0.11), while the correlation between the amplitude at 20 and 77 hPa is 0.81. This immediately suggests greater flexibility in tuning the period independently of the overall amplitude than in tuning the vertical structure of the QBO.

## 3 Survey of Sensitivity to Resolution, Dissipation, Resolved Waves, and Gravity Waves

Section 3.1 considers the sensitivity of the QBO to resolved processes, keeping the settings for the gravity wave scheme fixed. Section 3.2 then presents the sensitivity to the gravity wave scheme while keeping the numerics and boundary conditions fixed.

### 3.1 Sensitivity to Resolution, Dissipation, and Tropospheric Stationary Waves

**Figure 3:**
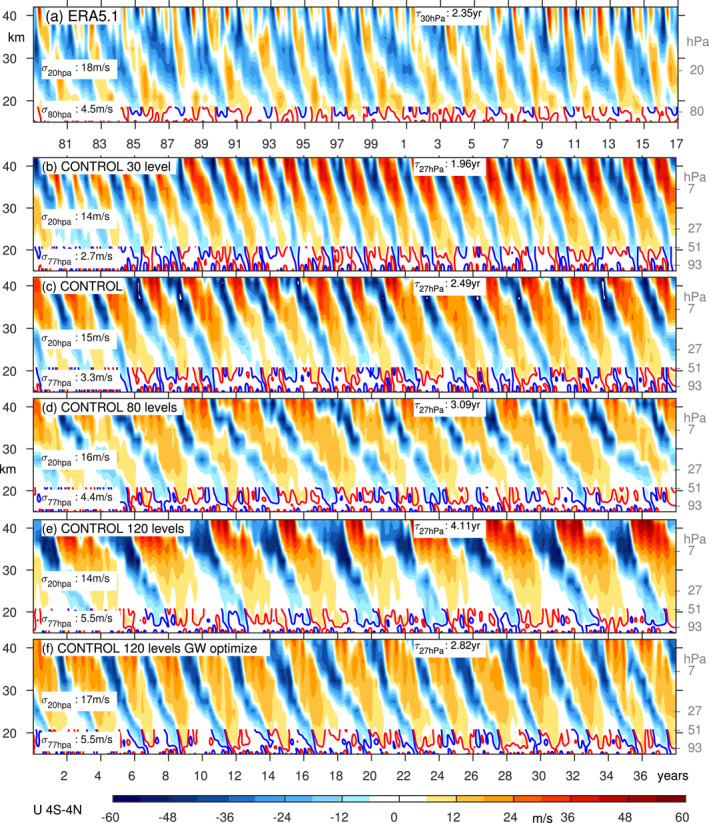
Zonal mean zonal wind from 4S‐4 N in (a) ERA5; Control at T42 with (b) 30, (c) 40, (d) 80, (e) 120 vertical levels, and (f) in a T42L120 run in which the gravity wave settings have been modified to improve the QBO periodicity. Specifically $B_{eq}$ is set to 6.3 mPa and $c_{w}$ in the tropics to 20 m/s. Each panel indicates the standard deviation of winds at 20 hPa and 77 hPa, and the period at 27 hPa. The contour interval is 6 m/s, and the 3 m/s contour is shown in blue and red in the lower stratosphere.

Figure 3a shows the QBO in the ERA5 reanalysis (Anstey et al., 2021; Hersbach et al., 2020; Pahlavan et al., 2021, the QBO is similar in other reanalyses) and Figure 3c shows the QBO at T42 with 40 vertical levels in our CONTROL. At this resolution, MiMA simulates a QBO similar to that observed: the period is slightly longer, but as shown later, relatively small changes to the settings in the model can lead to an exact match. The standard deviation of winds in the mid‐stratosphere is realistic, though it is under‐estimated lower in the stratosphere. Too weak QBO winds in the lower stratosphere is a common bias in QBOi and CMIP6 models (Bushell et al., 2020; Rao et al., 2020a; Richter et al., 2020), and the factors that lead to its amelioration will be discussed shortly.

**Figure 4:**
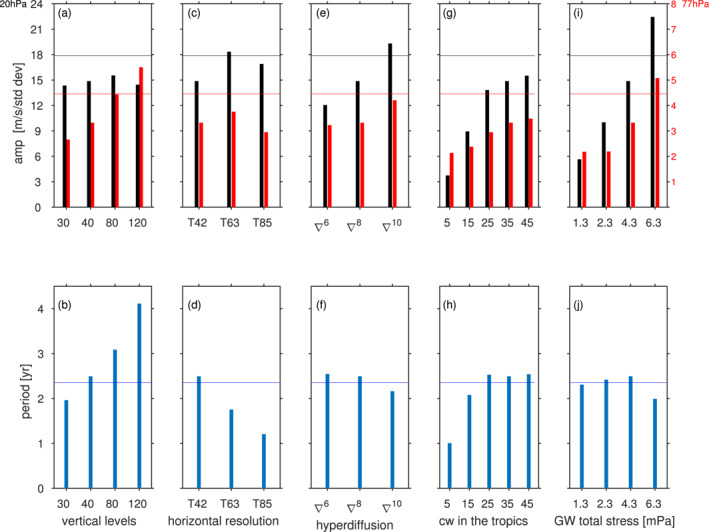
Summary of the sensitivities of the quasi‐biennial oscillation period and amplitude to (a and b) vertical resolution; (c and d) horizontal resolution; (e and f) hyperdiffusion order; (g and h) spectral width of the launched gravity waves in the tropics; (i and j) total gravity wave stress in the tropics. A horizontal line denotes the corresponding value from ERA‐5.

If the number of vertical levels is increased by a factor of 3, with the extra levels added in‐between the existing levels while the model lid is kept fixed for a vertical resolution of approximately 400 m in the lowermost stratosphere, the QBO period lengthens to 4.1 years (consistent with the lengthening of the period found in the model of Anstey et al. [2016]), while the standard deviation in the lowermost stratosphere, but not near 20 hPa, increases by more than ∼50% (Figure 3e; similar to the effect in the model of Geller et al. [2016]). A *decrease* in the number of vertical levels has an opposite effect (Figure 3b): a shorter period and a degradation in the standard deviation in the lowermost stratosphere; again the standard deviation in the mid‐stratosphere is unaffected. These changes are summarized in Figures 4a and 4b, which shows that both the standard deviation in the lowermost stratosphere and the period increase monotonically as vertical resolution is increased.

**Figure 5:**
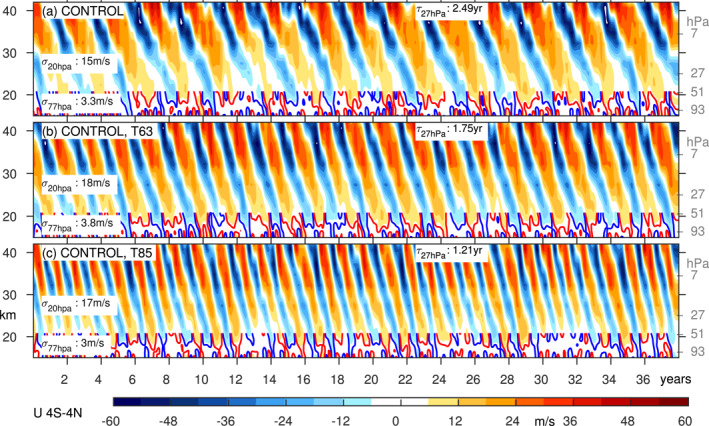
Zonal mean zonal wind from 4°S–4°N in (a) Control at T42 with 40 vertical levels (repeated from Figure 3c); (b) Control at T63 with 40 vertical levels; (c) Control at T85 with 40 vertical levels. Each panel indicates the standard deviation of winds at 20 hPa and 77 hPa, and the period at 27 hPa. The contour interval is 6 m/s, and the 3 m/s contour is shown in blue and red in the lower stratosphere.

If the horizontal resolution is increased to T63 or T85 (Figure 5, roughly equivalent to a grid of 1.9° or 1.4°), the period decreases to 1.75 and 1.2 years respectively. The amplitude increases for the T63 integration (consistent with Giorgetta et al. [2006]), but then decreases as the resolution is further increased to T85 (Giorgetta et al., 2006, did not consider T85 and we are not aware of any other relevant study). These changes are summarized in Figures 4c and 4d: the period decreases monotonically as horizontal resolution is increased, while the amplitude changes are less clear.

Models also differ in how they specify horizontal diffusion (Table 7 of Butchart et al. [2018]), and early modeling studies found sensitivity to this parameter (Takahashi, 1996). In our pseudo‐spectral model, the order n of the hyperdiffusion operator $\kappa \nabla^{n}$ governs the extent to which the diffusion is scale‐selective. Larger n leads to greater scale‐selectivity, and a smaller impact of diffusion on the large‐scale features. The net effect is that wavenumbers above the smallest resolved scale (i.e., 40 or 41 for T42) are damped more strongly if the damping order n is, say, 6 (i.e., $\nabla^{6}$ hyperdiffusion) than if n=10. The CONTROL hyperdiffusion is $\nabla^{8}$, and we explore sensitivity to n=6 and n=10 in Figures 4e and 4f; in all cases, we modify the hyperdiffusion coefficient κ such that the damping of the highest resolved wavenumber (42 at T42) is fixed so as to not impact the numerical stability of the model. Lowering *n* to 6 or raising it to 10 has a strong impact on the QBO amplitude: a lower value of n leads to a weaker QBO with an essentially unchanged period (Figure S1a in Supporting Information S1 and Figures 4e and 4f), while a larger value of n leads to a stronger QBO with a shorter period (Figure S1b in Supporting Information S1). This effect is due to the weaker damping on small‐scale resolved waves for a larger value of n.

Next, we explore sensitivity of the QBO to tropospheric stationary waves, while keeping other settings fixed. The stationary waves in CONTROL (both Kelvin and Rossby) compare favorably to those observed (Garfinkel et al., 2020a, 2020b; Garfinkel, White, et al., 2021), and as shown in Shamir et al. (2021) and Section 4.1, resolved tropical transient waves are reasonable as well. In order to quantify the impact of tropospheric stationary waves on the QBO, we remove land‐sea contrast, orography, and east‐west oceanic heat transport (as discussed in detail in Garfinkel et al. [2020a] and Garfinkel et al. [2020b]), while keeping the north‐south oceanic heat transport of Jucker and Gerber (2017), so that there are no zonal asymmetries in the lower boundary of the model. The resulting weakening of the stationary waves leads to a strengthening of the QBO by over 50% in both the mid‐stratosphere and lower stratosphere (zonally symmetric BC run in Figure 2 and Figure S1c in Supporting Information S1) and also to a slight decrease in the period.

Overall, the properties of the QBO are sensitive to the treatment of resolved waves while holding the gravity wave drag fixed. Specifically, the resolution, horizontal diffusion, and stationary waves all impact the QBO.

### 3.2 Sensitivity to Gravity Waves

We now turn our attention to the sensitivity of the QBO to the settings of the gravity wave scheme, taking CONTROL with T42 and 40 levels as the starting point. One of the tunable parameters in the Alexander and Dunkerton (1999) GW scheme (and indeed of most GW schemes) is the spectral width of the forced gravity waves ($c_{w}$ in Equation A1). If $c_{w}$ is decreased, then the gravity waves launched in the scheme will have a narrower range of phase speeds. The idealized models of Holton and Lindzen (1972) and Plumb (1977) predict that such a narrowing of launched phase speeds will lead to a decrease in the amplitude of the QBO winds. We now test this prediction in MiMA.

In CONTROL, $c_{w}=35$m/s, and we explore sensitivity to changing this parameter in Figures 4g and 4h and Figure S2 in Supporting Information S1. Note that $c_{w}$ is only changed from 10°S to 10°N (i.e., $c_{w}=35$m/s outside of the tropics) so as to not directly impact the representation of the midlatitude and polar stratosphere, and so minimally impact polar downwelling. The QBO is increasingly sensitive to $c_{w}$ if $c_{w}$ is less than around 25 m/s. For $c_{w}=5$m/s, the QBO essentially disappears, and for a $c_{w}=15$m/s the QBO standard deviation is little more than half of the standard deviation in the CONTROL integration and the period decreases. For $c_{w}$ of 25 m/s or higher, however, the change in the resulting QBO is relatively smaller: there is a saturation effect in the period and, to a lesser degree, in the amplitude in the mid‐stratosphere, even as the lower stratospheric amplitude continues to increase (Figures 4g and 4h).

An additional parameter of the gravity wave scheme in our model is $B_{eq}$, the total amplitude of the launched gravity wave stress in the tropics (see Equation A3); again, this is a common parameter of most GW schemes. In CONTROL, $B_{eq}$ is set to be identical to the global value $B_{0}$ (which is 0.0043 Pa), but this parameter is poorly constrained by observations and models often use higher or lower values (Figure 5 of Molod et al. [2012]). Figures 4i and 4j and Figure S3 in Supporting Information S1 assess sensitivity to the value of this parameter. Lowering $B_{eq}$ leads to a weakening of the QBO, as might be expected, with a slight decrease in the period. Increasing $B_{eq}$ leads to a stronger QBO and to a sharper decrease in the period. That a stronger $B_{eq}$ leads to a shorter period is consistent with Figure 1 of Geller et al. (2016), Table 2 of Rind et al. (2014), Figure 13 of Giorgetta et al. (2006), and Section 3.4 of Richter et al. (2014). We find, however, that the sensitivity of the period is non‐monotonic (Figures 4i and 4j).

A final parameter of the gravity wave scheme which is poorly constrained is the vertical level at which gravity waves are launched. The launch height in our setup is the sigma ($\frac{p}{p_{s}}$, where $p_{s}$ is the surface pressure) level closest to, but smaller than, 0.315, but other models launch at 100 hPa or even higher up (Anstey et al., 2016). Raising the launch level leads to a stronger QBO, and as an example we show in Figure S3e in Supporting Information S1 the QBO for a launch height of sigma = 0.15 and $c_{w}$ in the tropics of 25 m/s (run 11 on Figure 2, Figure S2c in Supporting Information S1). The QBO in Figure S3e in Supporting Information S1 has a larger standard deviation (run 17 on Figure 2) than in Figure S2c in Supporting Information S1 (which has a launch height at sigma = 0.315) in both the mid‐ and lower stratosphere as fewer gravity waves are filtered out before entering the stratosphere (Figure 2).

The sensitivities of the QBO to all of these model properties are summarized in Table 1. A wide range of “tuning knobs” are available, and while in our experiments the T42L40 QBO is closest to that observed outside of the lowermost stratosphere, this was the product of extensive tuning. A higher resolution version of the model could be tuned to also reproduce the QBO period and amplitude as well, a point we return to in the discussion.

**Table 1:** Summary of the Sensitivities of the Quasi‐Biennial Oscillation

	Period	Amplitude
Finer horizontal resolution	Faster	Small effect
Finer vertical resolution	Slower	Stronger but only in lowermost stratosphere
Higher hyperdiffusion power	Faster	Stronger
Adding tropospheric stationary waves	Small effect	Weaker
Wider gravity wave spectral width	Slower	Stronger, but effect saturates in mid‐stratosphere
Stronger gravity wave amplitude	Faster	Stronger
Higher gravity wave launch level	Small effect	Stronger

## 4 Making Sense of the Changes in Period and Downward Propagation to the Lowermost Stratosphere

Section 3 demonstrated that the QBO periodicity and downward propagation to the lower stratosphere are sensitive to a wide range of model parameters. We now seek to diagnose why. We focus on the metrics included in Figure 2, specifically the periodicity and the standard deviation at 77 hPa (i.e., in the lower stratosphere). This section considers not only the simulations discussed in Section 3 listed in Figure 2, but also simulations included in Garfinkel et al. (2020a) and Garfinkel et al. (2020b). As these facets of the QBO are intimately connected to the location of (pseudo‐)momentum fluxes associated with resolved and parameterized waves, we first consider the generation of resolved waves.

### 4.1 Generation of Resolved Waves

**Figure 6:**
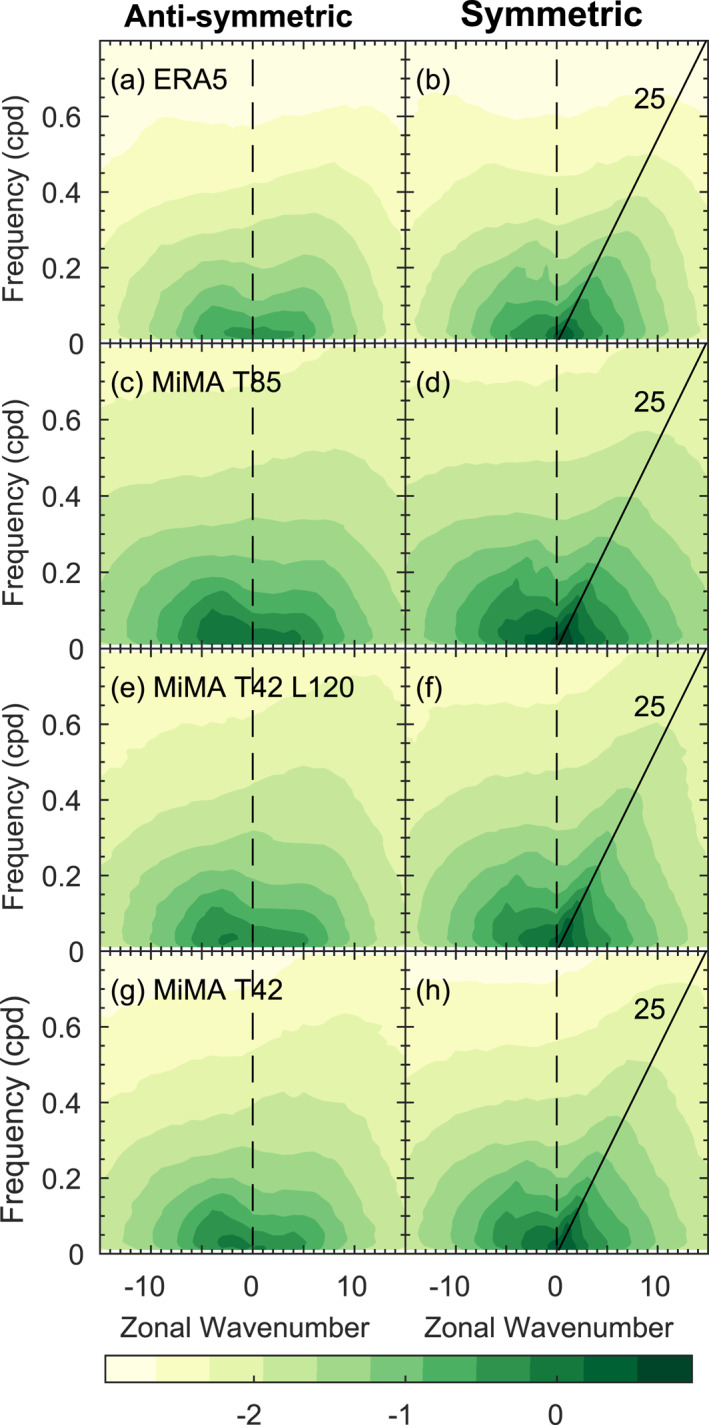
The logarithm base‐10 of the raw symmetric and anti‐symmetric spectrum of zonal wind at 200 hPa from 15°S to 15°N in (a and b) ERA5; (c and d) Control at T85 with 40 vertical levels; (e and f) Control at T42 with 120 vertical levels; (g and h) Control at T42 with 40 vertical levels.

The QBO is driven in part by transient waves that are well resolved at T42, and hence we show in Figure 6 the resolved waves in CONTROL and in the ERA‐5 reanalysis for zonal wind at 200 hPa from 15°S to 15°N. MiMA captures the redness of the spectrum in both time and wavenumber (Garfinkel, Shamir, et al., 2021; Shamir et al., 2021). It also exhibits enhanced power near the analytically predicted dry wave modes of Matsuno (1966), as is evident for Kelvin waves in the symmetric spectrum near a phase speed of 25 m/s. The spectrum is qualitatively similar in all resolutions in MiMA. There are differences between the observed spectrum and the spectrum in MiMA, however, and we focus on these differences in Figure S4 in Supporting Information S1. At all resolutions, the power is too strong except for symmetric ω−k combinations near the Madden‐Julian oscillation (k< 5 and low frequencies) which MiMA lacks. Note that Figure 6 and Figure S4 in Supporting Information S1 show the logarithm base‐10 of the power. Hence a difference of 0.5 in Figure S4 in Supporting Information S1 means $log_{10}\;\left(\text{MiMA}\right)-log_{10}\;\left(\text{ERA}5\right)=0.5$, or that MiMA has a factor of $10^{.5}∼3x$ more power. The bias in MiMA approaches a factor of three for ω−k combinations that are most energetic in Figure 6, however such a bias is well within the range of biases in the QBOi models evaluated by Holt et al. (2020).

**Figure 7:**
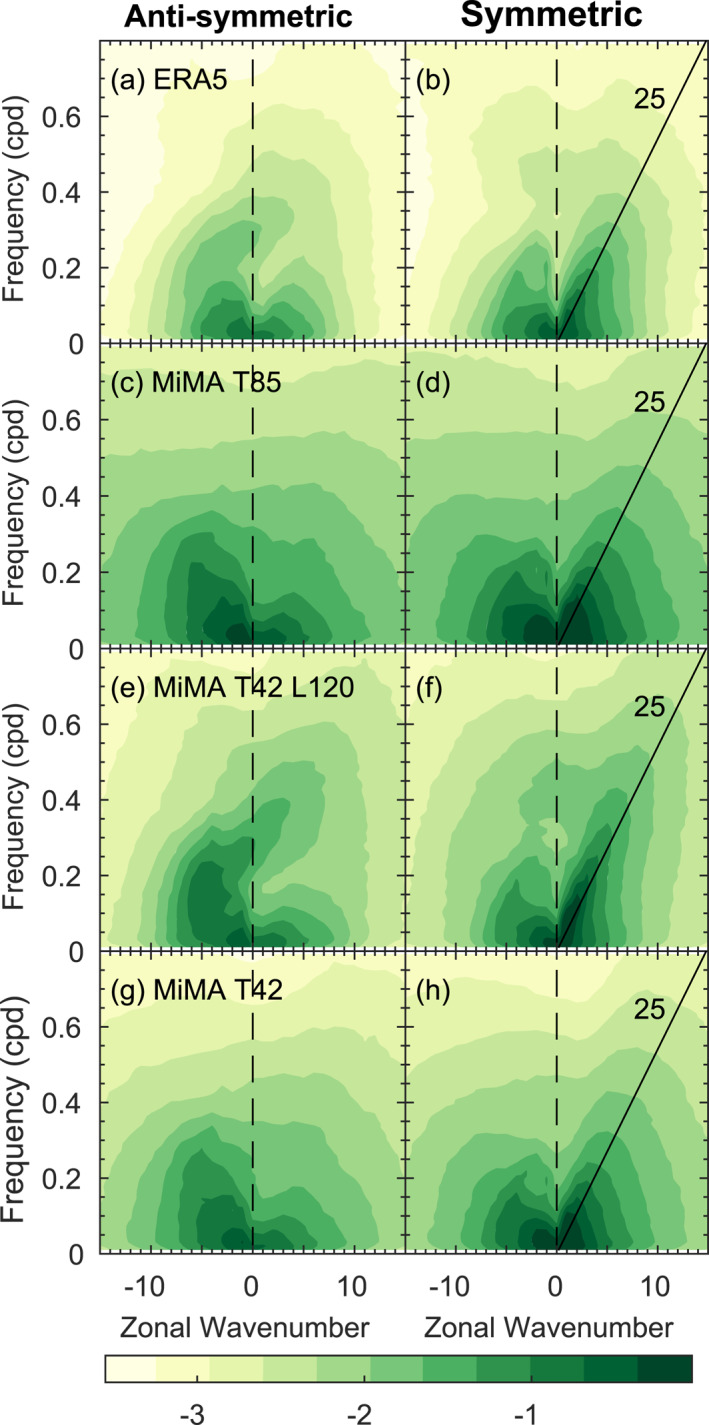
As in Figure 6 but for 77 hPa.

The spectrum closer to the base of the QBO is of more relevance for wave driving of the QBO. Hence we show the resolved wave spectrum at 77 hPa in Figure 7. It is evident that the simulations with 40 vertical levels struggle to simulate the mixed Rossby‐gravity mode (and to a lesser degree the Kelvin mode), while the simulation with 120 levels does capture these waves (Figure 7f vs. 7h for Kelvin, and Figure 7e vs. 7g for the mixed mode). Hence, while resolved waves in the troposphere are similar for different vertical resolutions, resolved waves higher up differ more strongly, though we note that integrations at all resolutions suffer from too much power at both 77 and 200 hPa (Figure S5 in Supporting Information S1). The implications for the QBO periodicity and downward propagation are considered in Section 4.2 and 4.3.

### 4.2 Explaining the QBO Period

We now attempt to quantify how resolved and parameterized waves drive the differences in the period of the QBO among these simulations. In order to do so, we first consider how the QBO is driven by these waves in CONTROL and then consider how this wave driving differs among the other experiments.

**Figure 8:**
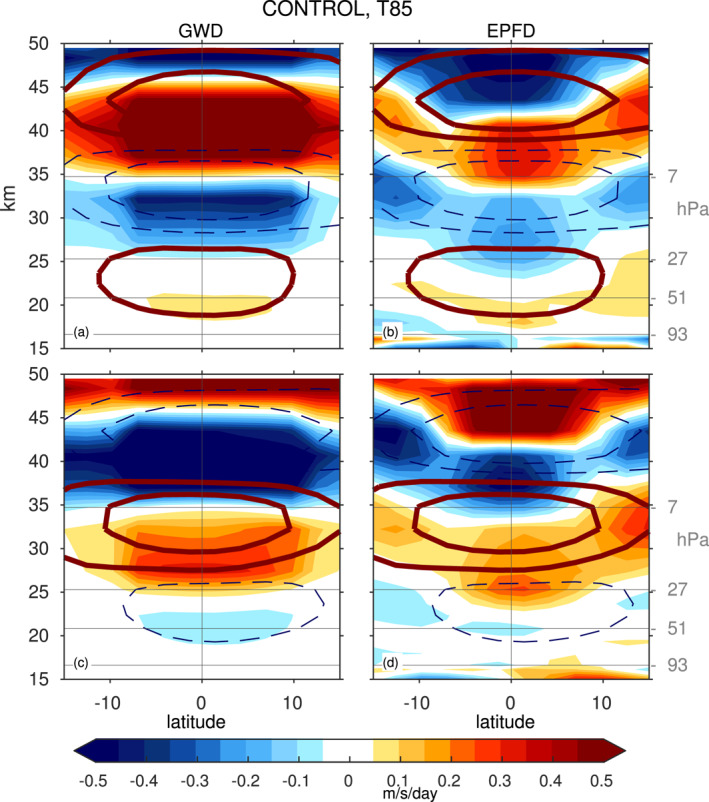
Forcing of winds by (left) parameterized gravity waves and (right) resolved waves for CONTROL at T85 for a quasi‐biennial oscillation (QBO) phase defined as wind anomalies at 41 hPa between (top) 10 and 15 m/s (i.e., WQBO) and (bottom) −10 and −15 m/s (i.e., EQBO). Results are similar for other resolutions (not shown). Shading shows the wind forcing, and contours the QBO zonal winds.

Taking CONTROL at T85 as an example, the top row of Figure 8 shows the zonal wind tendency due to parameterized GW drag and resolved waves (i.e., the Eliassen‐Palm flux divergence or EPFD) for a westerly QBO phase in the lower stratosphere (analogous to Figure 8 of Manzini et al. [2006], Figure 7 of Garcia and Richter [2019], and Figure 13 of Holt et al. [2020]), defined as winds at 40 hPa between 10 and 15 m/s stronger than climatology. The anomalous QBO winds are shown in solid brown and dashed blue. Similar to these previous modeling studies, parameterized gravity waves and EPFD from resolved waves are of similar importance in the lower stratosphere. Higher up, parameterized gravity waves dominate the forcing. The wave forcing is concentrated in the shear zones, and hence acts to propagate the anomalous QBO winds downward. The forcing is quantitatively similar but of opposite sign for the QBO phase with easterly winds in the lower stratosphere (bottom row of Figure 8).

**Figure 9:**
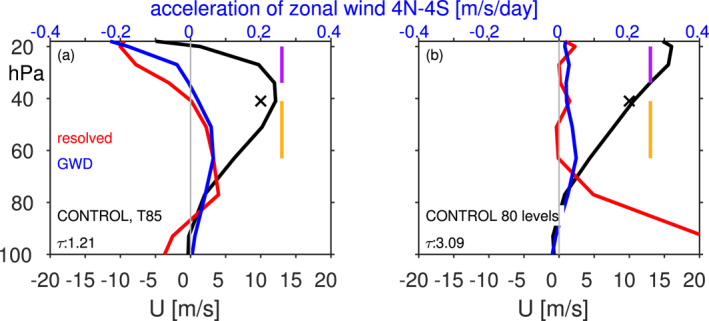
Quasi‐biennial oscillation (QBO) and its resolved and parameterized wave forcing in integrations with a relatively (left) fast period and (right) slow period for a WQBO composite in which anomalous zonal winds at 41 hPa must be between 10 and 15 m/s. The *x*‐axis for the QBO is shown on the bottom, and for the wave forcings on the top. Orange and purple vertical lines show regions averaged over for Figure 10. The QBO period is noted on the bottom left of each panel.

The forcing of the QBO and the QBO itself in Figure 8 is concentrated in the deep tropics, and we now distill the relative alignment of the QBO and its forcing by computing the deep‐tropical (4°S–4°N) averaged wave forcing due to resolved and parameterized gravity waves for this CONTROL T85 integration during the QBO phase with westerlies in the lower stratosphere (Figure 9a). The tropical zonal winds are shown in black. Both the resolved and parameterized waves are crucial in providing a westerly torque in the shear zone below the maximum westerlies, and hence allow for the downward propagation of the westerlies. Furthermore, both resolved and parameterized waves provide an easterly torque above the maximum westerlies. This vertically oriented dipole in momentum forcing supports the downward propagation of the QBO winds as the flux provided by waves is localized within the QBO shear zone.

Figure 9b is as in Figure 9a but for the T42L80 integration (whose period exceeds 3 years). In contrast to Figure 9a, the westerly torque is evident primarily in the lowermost stratosphere and not just in the shear zones, and the resolved wave forcing in particular peaks far from the shear zone. The net wave forcing within the shear zone is more effectively canceled out by the vertical advection term ($\bar{w^{*}}\frac{\partial u}{\partial z}$; not shown) leading to slow downward propagation and a longer period. The key point of Figure 9 is that for simulations with relatively short QBO periods (Figure 9a), the momentum flux convergence is concentrated in the shear zones, while for simulations with longer QBO periods (Figure 9b), the flux is spread out in the vertical over a much broader region. This effect is even more pronounced for resolved wave forcing than parameterized GW, and the net effect is that the wave forcing is less effective at propagating the QBO downwards due to a misalignment of the wave forcing with the maximum in wind‐shear.

**Figure 10:**
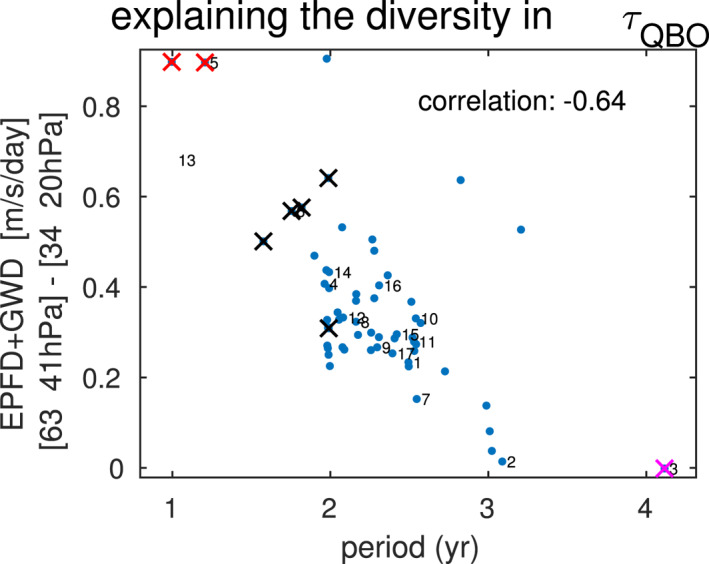
Relationship between quasi‐biennial oscillation periodicity and the difference in total wave driving on either side of the winds at 41 hPa (see orange and purple lines in Figure 9), for a WQBO composite in which anomalous zonal winds at 41 hPa must be between 10 and 15 m/s. Numbering of experiments follows Figure 2, and additional experiments performed as part of Garfinkel et al. (2020b) and Garfinkel et al. (2020a) are shown unnumbered for clarity. Black x‐es correspond to runs at T63, red x‐es to runs at T85, and magenta to runs with 120 levels.

In order to consider this effect for all simulations we have performed, we compute the difference in total wave forcing between the westerly shear zone (63 to 41 hPa, orange line on Figure 9) and the region above the QBO maximum (34 to 20 hPa, purple line on Figure 9). We then compare this differential zonal torque either side of 41 hPa to the QBO periodicity in Figure 10, with each simulation shown by a distinct marker. This figure includes not only the simulations discussed earlier in this study, but also the experiments included in Garfinkel et al. (2020a) and Garfinkel et al. (2020b). These two diagnostics are significantly correlated with each other (correlation of −0.64), whereby simulations with stronger westerly forcing in the westerly shear zone simulate a faster downward propagation and subsequently a shorter period. Results are similar if we average over a narrower or broader region on either side of the QBO wind maximum (not shown). The corresponding correlation for the easterly QBO regime is also statistically significant though weaker (correlation is 0.37; plot not shown). While a correlation does not imply causation and the wind profiles associated with a given QBO phase are not identical across different integrations, the overall effect is that a wave momentum forcing dipole with extrema on either side of the wind maximum will encourage downward propagation and a faster period.

The period of the QBO decreases when all tropospheric stationary waves are removed (Figure S1c in Supporting Information S1) in part due to a weakened Brewer‐Dobson Circulation (BDC) and hence weaker tropical upwelling. Indeed, the correlation between $\bar{w*}$ from 4°S to 4°N at 27 hPa with the QBO period for the integrations shown in Figure 10 is 0.34, whereby stronger upwelling leads to a longer period. While this relationship is statistically significant, the variance in periodicity associated with the residual circulation is much weaker than that associated with resolution, and hence the upwelling strength is not the determining factor for QBO period across all of our simulations. Indeed, if we focus on integrations at T42L40 with the gravity wave settings of CONTROL (and include all of the simulations of Garfinkel et al. [2020a] and Garfinkel et al. [2020b]), the correlation is essentially unchanged (correlation of 0.28).

### 4.3 Explaining the QBO Downward Propagation

**Figure 11:**
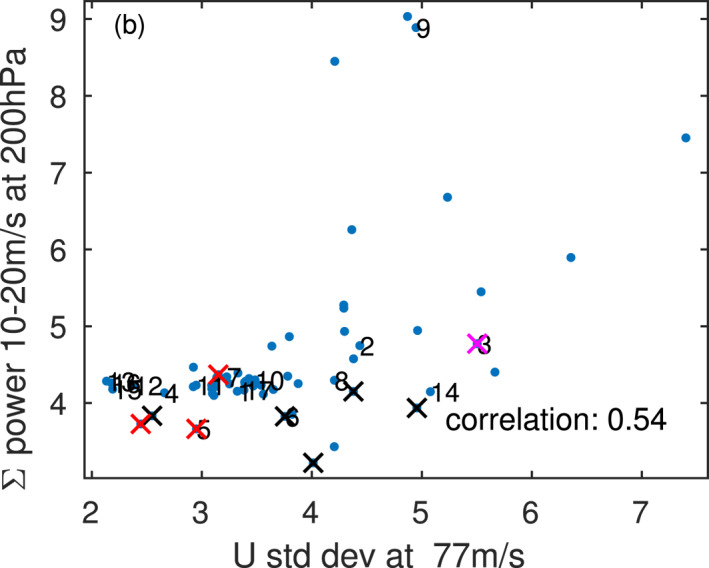
Relationship between quasi‐biennial oscillation standard deviation at 77 hPa and the resolved wave driving at 200 hPa between 10 and 20 m/s. The resolved wave driving in this range can be computed by summing over the appropriate spectral bins in, say, Figure 6. Numbering of experiments follows Figure 2, and additional experiments performed as part of Garfinkel et al. (2020b) and Garfinkel et al. (2020a) are shown unnumbered for clarity.

We now turn our attention to understanding the diversity of downward propagation into the lower stratosphere. Figures 4i and 4j and Figure S3 in Supporting Information S1 showed that a stronger flux of gravity waves leads to a larger amplitude QBO both at 20 and 77 hPa, and we now test the hypothesis that stronger resolved wave power also leads to a larger amplitude QBO. We quantify the role of resolved waves for the downward propagation using the total power at 200 hPa (below the base of the QBO) associated with variability between 10m/s and 20m/s for each simulation. We choose this range of power as we expect these waves to be most crucial for downward propagation in the lower stratosphere where winds are weak, though results are similar if we examine, say, 5m/sto15m/s or 5m/sto20m/s. Figure 11 compares the standard deviation of zonal winds at 77 hPa to this resolved wave power, with each simulation indicated with a marker. There is clearly a significant relationship between the two, and the correlation is 0.54; that is, a stronger wave forcing is associated with a larger amplitude QBO. The correlation for the easterly phase speeds between −10m/s and −20m/s is 0.35.

**Figure 12:**
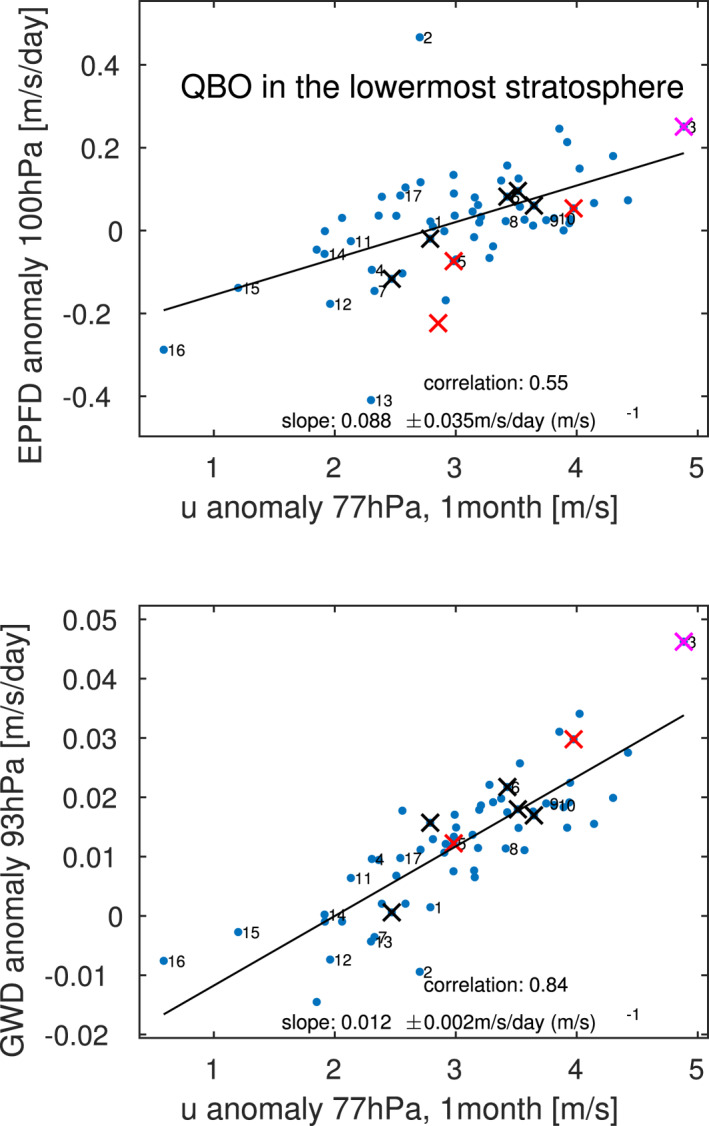
Relationship between winds in the shear zone below the quasi‐biennial oscillation (QBO) wind max and the wave driving lower down, for a WQBO composite in which anomalous zonal winds at 41 hPa must be between 10 and 15 m/s. Numbering of experiments follows Figure 2, and additional experiments performed as part of Garfinkel et al. (2020b) and Garfinkel et al. (2020a) are shown unnumbered for clarity.

An additional perspective on downward propagation can be obtained by considering the EPFD in the lowermost stratosphere during the QBO regime with strong westerly winds near 40 hPa, as we would expect enhanced resolved wave driving in the lowermost stratosphere to encourage downward propagation. Figure 12 considers this effect, and Figure 12a shows the relationship between winds in the shear zone below the QBO wind maximum and the resolved wave driving lower down, for a composite of events with WQBO winds in the lower stratosphere (composite definition as in Figure 9). Specifically, the ordinate shows the resolved wave EPFD near 100 hPa, while the absicca shows the wind anomaly at 77 hPa (in the shear zone) lagged by 1 month (EPFD is related to the time rate of change of zonal winds). There is clearly a strong relationship, and simulations with stronger resolved wave EPFD also simulate deeper propagation into the lowermost stratosphere with larger westerly wind amplitudes. Wave driving by gravity wave is also significantly correlated with downward propagation to the lowermost stratosphere (Figure 12b), however the regression coefficient for gravity waves is a factor of 8 smaller than that for resolved waves, so resolved waves seem to have a larger influence on the downward propagation in the lowermost stratosphere. Hence, we conclude that spread in the dissipation of resolved waves leads to the spread in the ability of the QBO to propagate downwards. This is consistent with the fact that only vertical resolution strongly impacts the amplitude of the QBO in the lowermost stratosphere.

## 5 Discussion and Conclusions

The QBO is the dominant mode of variability in the tropical stratosphere, and while the wind anomalies are confined to the tropics, it impacts the atmospheric circulation and composition globally through a variety of mechanisms. Most models participating in various model intercomparison projects have failed to simulate the QBO, and even the recent CMIP6 and QBOi models that succeed in simulating a QBO‐like oscillation suffer from a wide range of biases in the QBO behavior. The goal of this work is to provide a “cookbook” as to the sensitivities of the QBO to a range of processes, so as to enable modeling groups to more efficiently hone their efforts toward improving properties of the QBO.

Table 1 and Figure 4 summarize the sensitivities of the QBO. Finer horizontal resolution is shown to lead to faster QBO downward propagation. Finer vertical resolution is shown to lead to a longer period (if the GW settings are unchanged) and to an increased amplitude in the lowermost stratosphere. An increase in the order of numerical hyperdiffusion leads to a shorter period and a stronger amplitude. Enhancing tropospheric stationary waves leads to a weaker amplitude. A wider parameterized gravity wave spectral width at the source level leads to a slower and a stronger QBO, but the amplitude effect saturates in the mid‐stratosphere. A stronger gravity wave stress at the source leads to a faster and stronger QBO. Launching the gravity wave at a higher level leads to a stronger QBO. While these sensitivities appear robust in our modeling framework, we suspect that they can only provide qualitative guidance for other models while the quantitative details may vary. For example, the regression coefficient between changes in the gravity wave stress at the source and the QBO standard deviation likely depends on the specific gravity wave parameterization implemented in a given model.

These sensitivities are shown to result from the details of the resultant wave‐driven zonal wind torque in the stratosphere. The period of the QBO is sensitive to the relative wave‐driven torque directly below versus directly above the QBO wind maximum, and models that simulate a dipole in total wave‐driven torque, with acceleration below and deceleration above, simulate a faster period (Figure 10). The amplitude of the QBO is shown to be related to the magnitude of the wave momentum flux with relevant phase speeds that can reach the stratosphere. More wave momentum flux, whether parameterized gravity or resolved, leads to a stronger QBO in the mid‐stratosphere (Figures 11 and 12).

Many models suffer from a too weak amplitude bias in the lowermost stratosphere. Of the various parameters that can be tuned, the only “fix” we identified that does not simultaneously increase the amplitude in the mid‐stratosphere was to increase vertical resolution. This result is consistent with Giorgetta et al. (2006), Geller et al. (2016), and Anstey et al. (2016, among others) who also find sensitivity of the QBO to vertical resolution. There are other ways of increasing the amplitude at 77 hPa and simultaneously the amplitude higher up, but then a bias in the lower stratosphere is replaced with a bias in the mid‐stratosphere; the only way we found to independently modify the amplitude in the lower stratosphere separately from the mid‐stratosphere is via vertical resolution.

Another bias that is only “fixed” with increased resolution is the duration of the westerly regime as compared to the easterly regime. In observations, the easterly regime persists for longer at and above 20 hPa while the westerly regime persists for longer near 77 hPa (Figure 3a). This asymmetry is represented in the 120 level run (Figure 3e), but not in any of the L40 runs (Figure 3c and Figures S1–S3 in Supporting Information S1). The amelioration of these biases is likely related to the ability of Kelvin waves to drive the westerly regime in the lowermost stratosphere if 120 levels are used, but these Kelvin waves are poorly represented with 40 levels (Figures 6 and 7). Specifically, a strong Kelvin wave climatology when 120 levels are used appears to result in a near‐persistent layer of westerlies in the lower stratosphere that resists the downward propagation of the next easterly phase, and shortens the easterly phase when it finally does penetrate.

In contrast, in the mid‐stratosphere the wave forcing is more dependent on the parameterized GW, and thus the mid‐stratospheric properties of the QBO can be modified by tuning the GW stress. We now demonstrate explicitly how retuning the gravity wave parameterization can lead to an improved QBO, taking the T42L120 CONTROL run as an example. Recall that this integration simulates a realistic downward propagation to the lowermost stratosphere and a reasonable amplitude, but the period is 4.1 years and thus too long. Our goal is to retune the gravity waves so as to lower the period while minimally modifying the amplitude. Specifically, we set $B_{eq}$ to 6.3 mPa and $c_{w}$ in the tropics to 20 m/s; both of these changes should lead to a reduction in the period, while their impacts on the amplitude should mostly cancel out (Figure 4). The resultant QBO is shown in Figure 3f (as compared to Figure 3e). It is clear that the QBO period is substantially improved, even as the amplitude is generally the same. This experiment demonstrates how the QBO cookbook provided in this study can be used to more efficiently tune the QBO.

The QBO in MiMA does not converge numerically. Namely, increasing the resolution does not lead to a QBO that is more realistic as compared to observations. However, the total resolved wave flux, and more importantly the details of where this flux deposits momentum, differs depending on the resolution, and the QBO is sensitive to the total flux, not just the resolved flux. This highlights the fact that the GW parameterization in models must be scale‐aware, and that the properties of parameterized GW must be carefully adjusted for each resolution.

When run with 40 vertical levels, the vertical resolution in the lower stratosphere and tropical tropopause layer is approximately 1.2 km. Previous studies using models with such a coarse resolution typically failed to simulate a QBO (Anstey et al., 2016; Geller et al., 2016; Giorgetta et al., 2006; Richter et al., 2014), though Rind et al. (2014) note that such a coarser vertical resolution still enabled the spontaneous generation of a QBO, but it failed to propagate down to the lower stratosphere. We speculate that we nevertheless succeed in simulating a QBO because the resolved wave power spectrum in MiMA is stronger than observed at 200 hPa (Figure S4 in Supporting Information S1) and importantly also at 77 hPa (Figure 7 and Figure S5 in Supporting Information S1), and so the resolved wave forcing of the QBO is still reasonable (as quantified in Section 4).

A notable exception to the general tendency of models with poor vertical resolution to fail to simulate a QBO‐like oscillation comes from the studies of Yao and Jablonowski (2013) and Yao and Jablonowski (2015). They studied the spontaneous development of a QBO‐like oscillation in a dry dynamical core with no convection or gravity wave scheme. Their model nevertheless supported a QBO‐like oscillation, though the period was too long and the downward propagation did not extend to the lower stratosphere. They found that a spectral dynamical core supported this QBO‐like oscillation more than a finite volume dynamical core, and indeed our configuration of MiMA uses a spectral dynamical core.

The observed QBO is often linked with the semi‐annual oscillation in the mesosphere and upper stratosphere. Our model does not simulate a semi‐annual oscillation, likely due to the model lid near 70 km, and the requirement that the gravity wave scheme smoothly deposits momentum in the top model levels (as discussed in Appendix A).

None of our simulations simulate disruptions as extreme as those that have occurred in the past five years (e.g., near 2016 in Figure 3a), though the simulations with weak QBOs occasionally skip a particular phase and instead simulate a prolonged, for example, westerly phase (see the $B_{eq}=0.0023$ simulation near year 30 in Figure S3 in Supporting Information S1). Hence a disruption can arise spontaneously if there is relatively weak gravity wave flux leaving the troposphere, even as no external perturbations are imposed in the troposphere. While such a mechanism may not be relevant for the disruption in 2015/2016 when wave activity was anomalously strong (Kang et al., 2020), a weakening of the QBO under climate change (Kawatani & Hamilton, 2013; Rao et al., 2020c) may make it more susceptible to disruptions.

Overall, this study shows that a wide range of parameters affect the QBO, and hence we expect that biases in, for example, QBO strength or periodicity can be “fixed” in a comprehensive model by carefully adjusting these parameters in parallel. This effect is demonstrated in Figure 3f: Figure 3f shows a remarkably realistic QBO (certainly better than that in many of the CMIP models considered by Richter et al. [2020], Rao et al. [2020a], and Rao et al. [2020b]), particularly in terms of its penetration into the lower stratosphere, obtained by enhancing the vertical resolution and adjusting the gravity wave parameterization source spectrum.

## Supporting information

Supporting Information S1

## Data Availability

The updated version of MiMA used in this study including the modified source code and example name lists to reproduce the experiments can be downloaded from https://github.com/ianpwhite/MiMA/releases/tag/MiMA-ThermalForcing-v1.0beta (with DOI: https://doi.org/10.5281/zenodo.4523199). It is expected that these modifications will also eventually be merged into the main MiMA repository which can be downloaded from https://github.com/mjucker/MiMA.

## Appendix A Implementation of a Gravity Wave Scheme in a Model of an Idealized Moist Atmosphere (MiMA)

Gravity waves have important global effects on the circulation, temperature structure, and composition of the atmosphere, but occur on spatial scales that are too fine to be resolved by nearly all general circulation models (Alexander et al., 2010). Gravity waves carry momentum and energy vertically in the atmosphere, and they are an important forcing term in the stratospheric momentum budget. Models must parameterize these forcing terms using information on the larger‐scale wind and stability fields. Most gravity wave schemes share a few common attributes: a series of waves with various possible combinations of the ground‐relative phase speed and horizontal wavenumber are launched, and the dissipation of the waves as a function of height is based on the concepts of “breaking” (Lindzen, 1981) due to the presence of critical lines, and “saturation” (Dunkerton, 1989; Fritts, 1984), as density decreases and gravity wave amplitude grows. We parameterize gravity waves following Alexander and Dunkerton (1999), Donner et al. (2011), and Cohen et al. (2013), and while the criteria for breaking and dissipation of waves is left unchanged, we have modified the properties of the wave source. We now document these changes.

A key parameter in any parameterization of gravity waves is the distribution of stress across phase speeds, and we thus repeat the treatment of this in the parameterization of Alexander and Dunkerton (1999) (their Equation 17):

(A1)
$$B_{0}\;(c)=\mathrm{sgn}\left(\hat{c}\right)B_{m}\exp\left[-{\left(\frac{c-c_{0}}{c_{w}}\right)}^{2}\ln2\right]$$

Here *c* is the ground‐relative phase speed; $c_{0}$ is the phase speed with maximum flux magnitude $B_{m}$, and in all experiments in this study $B_{m}=0.4\;m^{2}/s^{2}$; $c_{w}$ is the half‐width at half‐maximum of the Gaussian (35m/s in all integrations poleward of 10°S and 10°N, and 35m/s in the tropics as well unless specified otherwise); and $\hat{c}$ is the intrinsic phase speed at source level. The source level is set at 315 hPa in the tropics (following Donner et al., 2011) unless otherwise specified. The spectral resolution for the phase speed bins is 2 m/s, and the tropical wave spectrum is set to be symmetric about the zonal wind at the source level ($c_{0}$ is set to the zonal wind), for all integrations shown in this study.

$B_{0}\;(c)$ represents the gravity wave amplitude during an active wave event, however gravity waves are by their very nature intermittent. The parameterization of Alexander and Dunkerton (1999) handles this intermittency by a separate parameter $F_{S0}$ which is intended to represent the long‐term average of momentum flux integrated across all phase speeds. $F_{S0}$ and $B_{0}\;(c)$ are related by an intermittency factor ϵ following Equation 19 of Alexander and Dunkerton (1999) as

(A2)
$$\epsilon =\frac{F_{S0}\Delta c}{\bar{\rho_{o}}\sum_{c}|B_{0}\left(c\right)|\Delta c}$$

The value of $F_{S0}$ in many GW parameterizations is not constant in latitude (Anstey et al., 2016; Donner et al., 2011; Molod et al., 2012), and we explore the importance of latitudinal dependence in $F_{S0}$ as described in Equation A3:

(A3)
$$F_{S0}\left(\phi \right)=\left\{\begin{array}{cc} Bt_{0}\;+\;0.5Bt_{SH}\left(1.+\tanh\left(\frac{\phi -\phi_{0s}}{\delta \phi_{s}}\right)\right)\; & ,\;\phi \leq \phi_{0s}\\ Bt_{0}\;+\;\frac{Bt_{eq}-Bt_{0}}{\phi_{0s}-\delta \phi_{s}}\left(\phi_{0s}-\phi \right)\; & ,\;\phi_{0s}\leq \phi <\delta \phi_{s}\\ Bt_{eq}\; & ,\;\delta \phi_{s}\leq \phi \leq \delta \phi_{n}\\ Bt_{0}\;+\;\frac{Bt_{eq}-Bt_{0}}{\phi_{0n}-\delta \phi_{n}}\left(\phi_{0n}-\phi \right)\; & ,\;\delta \phi_{n}<\phi \leq \phi_{0n}\\ Bt_{0}\;+\;0.5Bt_{NH}\left(1.+\tanh\left(\frac{\phi -\phi_{0n}}{\delta \phi_{n}}\right)\right)\; & ,\;\phi \geq \phi_{0n} \end{array}\right.$$

In CONTROL, $Bt_{0}=0.0043$Pa, and $Bt_{eq}=Bt_{0}=0.0043$, such that the same stress is imposed in both the tropics and subtropics, but we explore sensitivity to $Bt_{eq}$. Additional stress is included in midlatitudes and subpolar latitudes by setting $Bt_{NH}$ = 0.0035 Pa and $Bt_{SH}=0.0035$Pa; this extra drag helps to keep the polar vortex from becoming too strong. Note that we do not include any orographic gravity wave drag in our model setup. Finally, $\phi_{0n}=15$, $\phi_{0s}=-15$, $\delta \phi_{n}=10$, $\delta \phi_{s}=-10$ specify the meridional extent of the QBO, and are also unchanged in all of our experiments. This functional form loosely follows a similar form in the GEOSCCM model and MERRA‐2 reanalysis (Figure 5 of Molod et al. [2012]) and the Canadian Middle Atmosphere Model (CMAM, Anstey et al., 2016). The net effect of this change is that the intermittency factor ϵ is made a function of latitude, and specifically gravity waves are more frequently present in midlatitudes, and also in the tropics if $Bt_{eq}$ is larger than $Bt_{0}$.

An additional change made from the configuration in Alexander and Dunkerton (1999) and Cohen et al. (2013) is that the momentum associated with gravity waves that would leave the upper model domain is deposited evenly in the levels above 0.85 hPa in order to conserve momentum. (There are three such levels when the model is run with 40 total levels.) This avoids any complications noted by Shepherd and Shaw (2004) and Shaw and Shepherd (2007) associated with non‐conservation of momentum. Note that Cohen et al. (2013) inserted this momentum evenly in the levels above 0.5 hPa. No sponge layer is included in the model. This requirement, coupled with the lid near 70 km, likely kills the semi‐annual oscillation in MiMA.
