# Supplementary material for: A QBO Cookbook: Sensitivity of the Quasi‐Biennial Oscillation to Resolution, Resolved Waves, and Parameterized Gravity Waves
Source: J Adv Model Earth Syst. 2022 Mar 22;14(3):e2021MS002568. doi: 10.1029/2021MS002568 (PMC9286580; doi:10.1029/2021MS002568)
Supplement: Supplementary file 1 — Supporting Information S1 [file JAME-14-0-s001.pdf]

Supporting Information for "A QBO cookbook: Sensitivity of the Quasi-Biennial Oscillation to resolution, resolved waves, and parameterized gravity waves"

Chaim I. Garfinkel [1], Edwin P. Gerber [2], Ofer Shamir[1], Jian Rao[1,4], Martin Jucker[3], Ian White[1], Nathan Paldor[1]

[1]Fredy and Nadine Herrmann Institute of Earth Sciences, Hebrew University, Jerusalem, Israel.

[2]Courant Institute of Mathematical Sciences, New York University, New York, USA

[3]Climate Change Research Centre and ARC Centre of Excellence for Climate Extremes, University of New South Wales, Sydney, Australia

[4] Key Laboratory of Meteorological Disaster, Ministry of Education (KLME) / Joint International Research Laboratory of Climate and Environment Change (ILCEC) / Collaborative Innovation Center on Forecast and Evaluation of Meteorological Disasters (CIC-FEMD), Nanjing University of Information Science and Technology, Nanjing 210044, China

Contents of this file

1. Figures S1 to S5

Introduction

The supplemental material contains additional three figures analogous to Figure 3 in the main body but for additional integrations. Supplemental Figures 4 and 5 compares the resolved wave power spectrum in our model to reanalysis data.

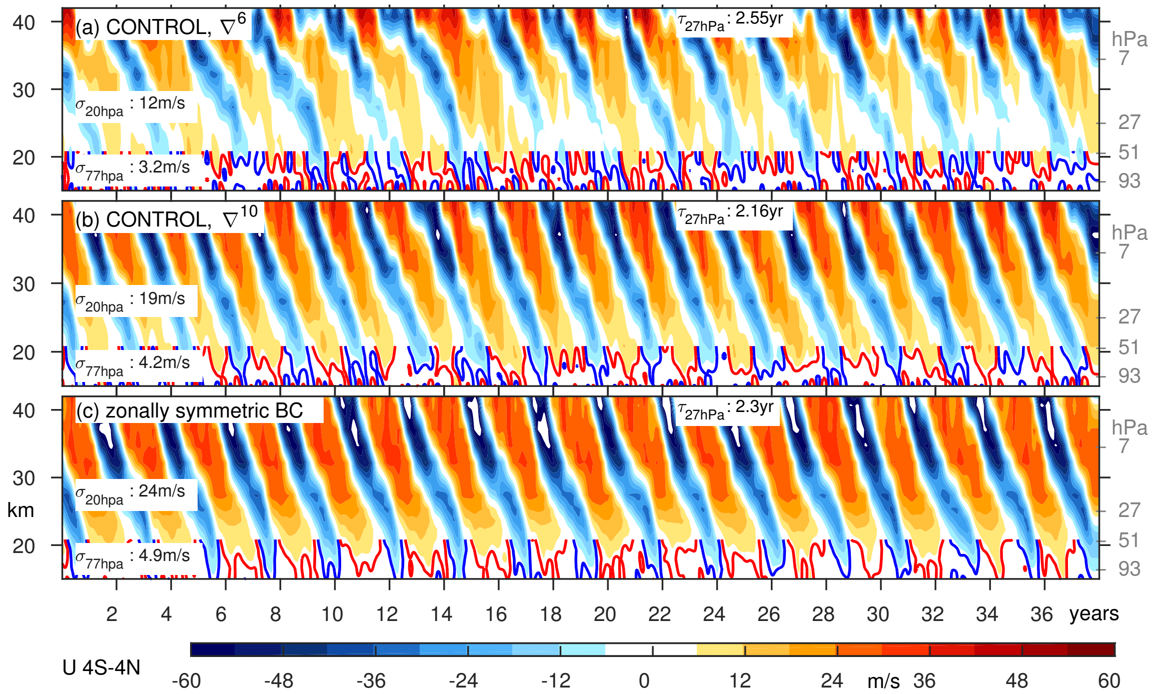

Figure S1. As in Figure 3 but exploring the role of hyperdiffusion of small scale resolved waves and stationary waves.

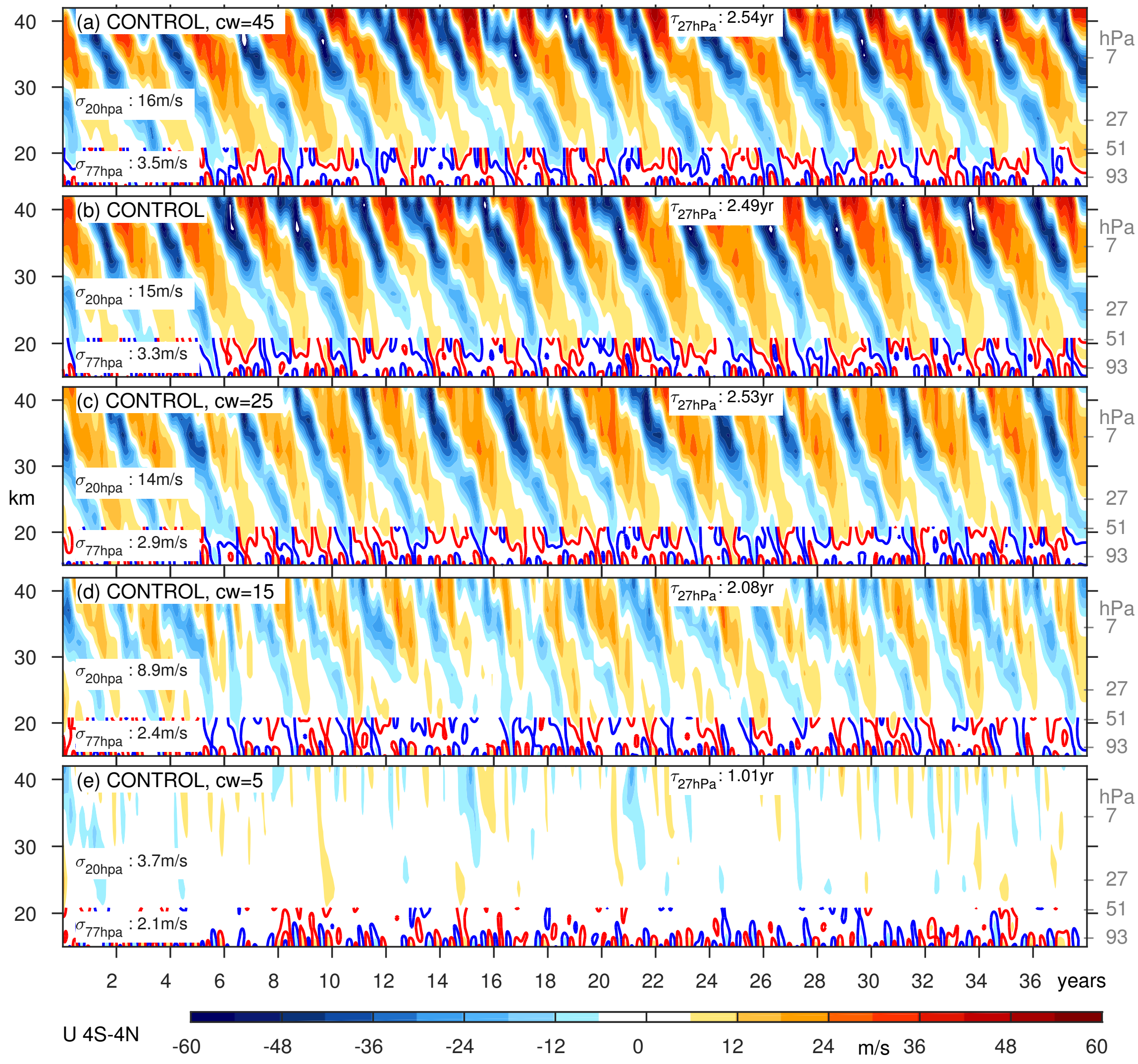

Figure S2. As in Figure 3 but exploring the role of the spectral width of the parameterized gravity wave forcing, with a spectral width in the tropics of (a) 45m/s; (b) 35 m/s; (c) 25m/s; (d) 15m/s; (e) 5m/s.

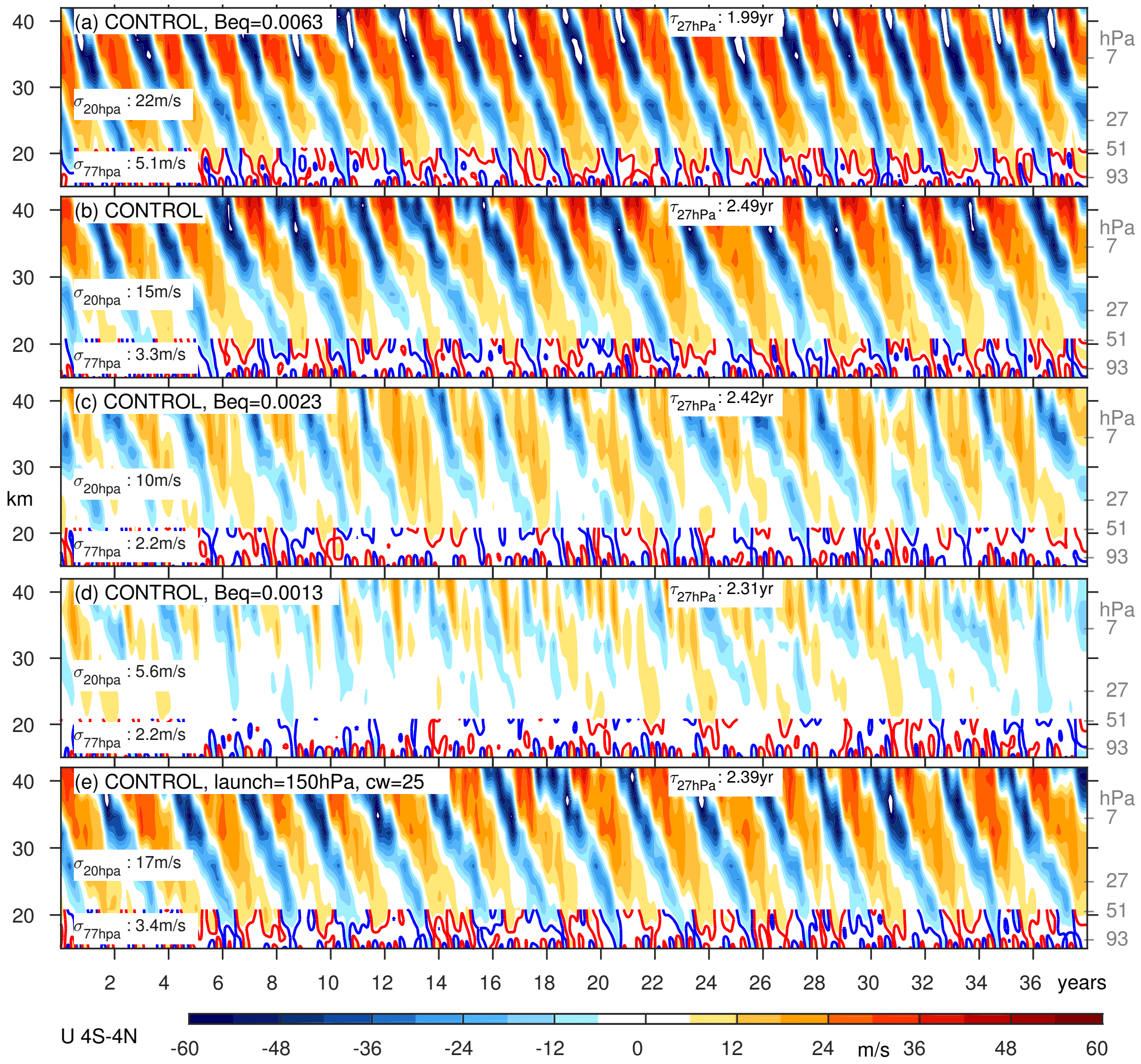

Figure S3. As in Figure 3 but exploring the role of the (a-d) amplitude of the parameterized gravity waves and (e) raising the launch height.

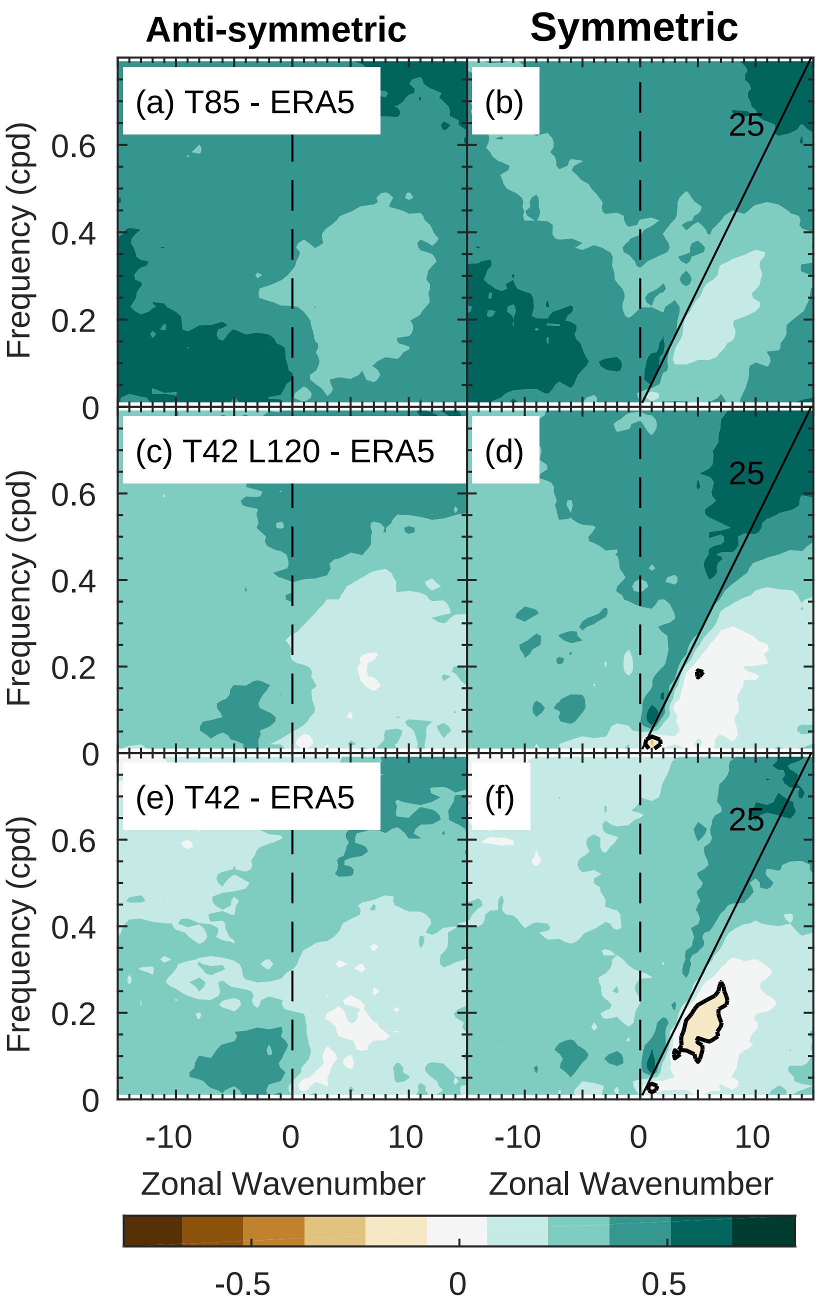

Figure S4. Difference of the log_{10} of the raw spectrums in MiMA shown in Figure 5c-h (for 200hPa) with that in ERA5 in Figure 5ab.

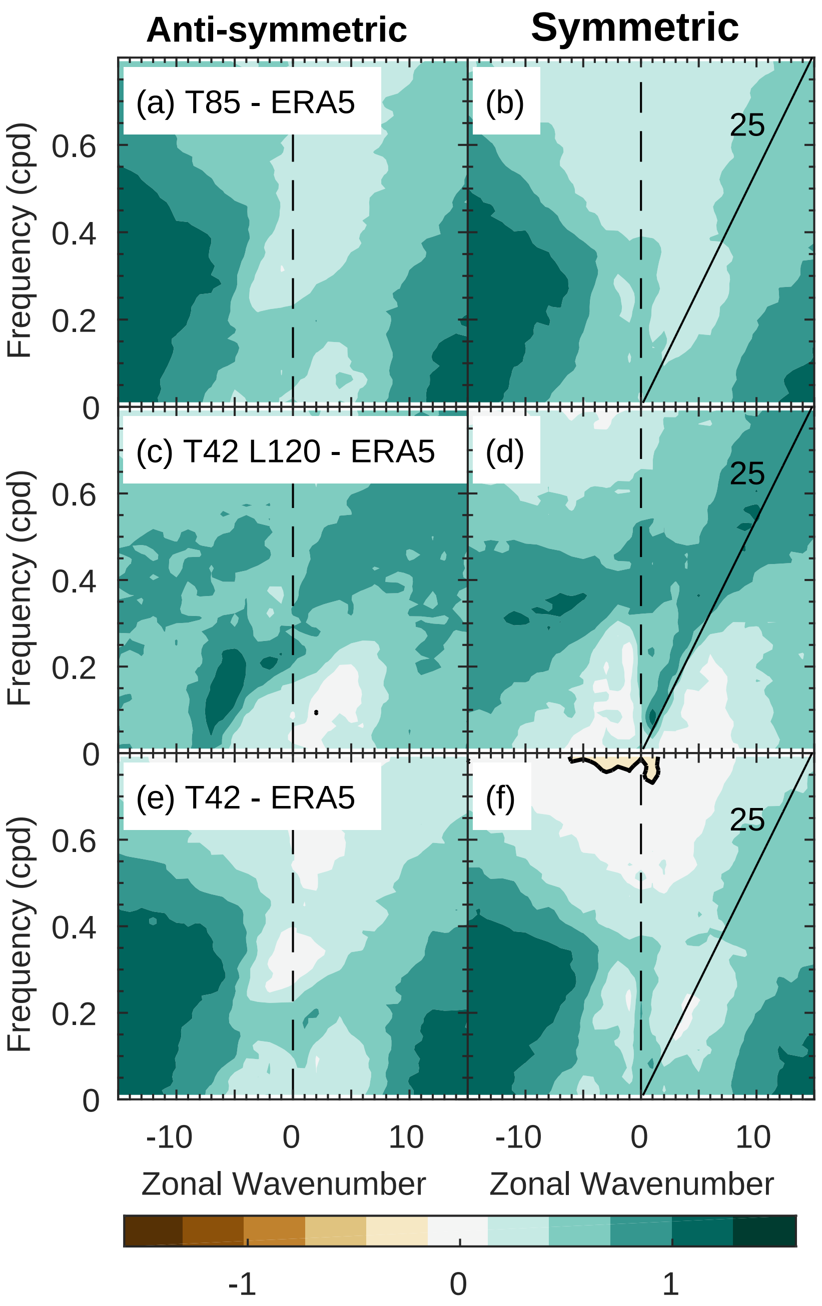

Figure S5. Difference of the log_{10} of the raw spectrums in MiMA shown in Figure 6c-h (for 77hPa) with that in ERA5 in Figure 6ab.
